# Cigarette or E-Cigarette Use as Strong Risk Factors for Heated Tobacco Product Use among Korean Adolescents

**DOI:** 10.3390/ijerph17197005

**Published:** 2020-09-24

**Authors:** Jun Hyun Hwang, Dong Hee Ryu, Inho Park, Soon-Woo Park

**Affiliations:** 1 Department of Preventive Medicine, Catholic University of Daegu School of Medicine, Daegu 42472, Korea; pmdr213@cu.ac.kr (J.H.H.); ryudh@cu.ac.kr (D.H.R.); 2 Department of Statistics, College of Natural Sciences, Pukyong National University, Busan 48513, Korea; ipark@pknu.ac.kr

**Keywords:** heated tobacco products, heat-not-burn, cigarettes, e-cigarettes

## Abstract

Heated tobacco products (HTPs) were first introduced in Korea in June 2017. This study examined the prevalence of current HTP use among Korean adolescents and its association with conventional cigarette (CC) or electronic cigarette (EC) use. The study analyzed nationally representative data (the 2019 Korea Youth Risk Behavior Web-based Survey) from a sample of 57,303 Korean students from grades 7–12. Multinomial logistic regression models were designed to evaluate the association between EC or CC use and HTP use. A total of 2.6% of respondents were current HTP users and 95.9% of them were dual or triple users of CC or EC. The likelihood of HTP use was higher among current CC or EC users and highest among dual users. When the association between each tobacco product and current HTP use was analyzed, the dual use tendency of HTPs and other products steadily increased with the increase of CC or EC smoking frequency. Adolescents who use ECs and/or CCs are likely to use HTPs. Thus, HTPs could be a new public health concern for adolescents in terms of dual or triple use patterns of CC or EC.

## 1. Introduction

Heated tobacco products (HTPs) are electronic devices that generate nicotine-containing aerosol without combustion [1]. In 2014, Philip Morris International (PMI) launched an HTP named IQOS^®^ in Italy and Japan [1]. Today, IQOS^®^ is sold in approximately 50 countries [2]. In October 2019, the Food and Drug Administration (FDA) began permitting the sale of IQOS^®^ through a premarket tobacco product application pathway in the United States [3]. Since the emergence of IQOS^®^, new products like glo^®^ (by British American Tobacco) and lil^®^ (by KT&G Corporation) have flooded the market. Tobacco companies market HTPs as a less harmful alternative to conventional cigarettes (CCs) [4]. During the modified risk tobacco products (MRTP) approval process, in July 2020, the FDA authorized marketing of IQOS^®^ as an MRTP without fully reviewing sufficient data of their long-term health risks [5,6]. This authorization for IQOS^®^ could be withdrawn depending on results from post-market surveillance and subsequent studies [6]. In addition, the FDA denied the risk modification order to significantly reduce harm and the risk of tobacco-related disease to individual tobacco users, one of two types of MRTP [6]. Therefore, to date, the debate over the health risks of HTPs is ongoing.

Although some tobacco companies, such as PMI, have publicly expressed their opposition to HTP use by adolescents [7], young people are easily exposed to HTP marketing strategies through social media [8]. Additionally, since HTPs are appealing to adolescents [9], it is necessary to investigate HTP use among adolescent. In Japan and Korea, limited research regarding HTP use among adults has been conducted and few studies have addressed HTP use among adolescents. Researchers have noted that most adult HTP users are dual users of CC and HTP, suggesting that HTPs are a complement to CC, not an alternative as claimed by tobacco companies [10,11,12]. Studies focused on HTP use by adolescents have assessed primarily their awareness or interest in HTPs. A Japanese study included 881 adolescents (10.7% of study participants) aged 15 to 19 years. However, no specific analysis regarding factors associated with HTP use for this age group was conducted. In addition, representation for the study is questionable because only online respondents were considered [12]. A study conducted in Korea revealed that 2.9% of Korean adolescents (men: 4.4%, women: 1.2%) were HTP ever users, most of whom were dual users of HTP and CC or triple users of HTP, CC, and electronic cigarettes (EC). However, due to the unavailability of data, it was not possible to assess current or former HTP use status and related factors in the aforementioned study [13]. Dual or poly-tobacco use among adolescents is a public health problem [14]. In research conducted with US adolescents, the probability of HTP use among EC users was reported to be higher than among cigarette smokers [15]. Therefore, in evaluating the related factors of HTP use, it is important to evaluate not only CC use but also EC use.

In Korea, the HTP market has experienced a rapid growth since the launch of IQOS^®^ (in June 2017), along with other products, such as lil^®^ and glo^®^. HTP use among Korean young adults reached 4.3% within one year of IQOS^®^’ release [10], surpassing that of Japanese young adults [12]. Moreover, the total sales of CCs decreased from 304 million packs to 276 million packs from June 2017 to September 2019, while sales of HTPs increased from 1.7 million packs to 30 million [16]. This suggests a significant change in the smoking behaviors of Korean adolescents.

The present study examined nationally representative data to reveal the prevalence of HTP use among Korean adolescents and the relationship of HTPs with the use of other tobacco products (CCs and ECs).

## 2. Materials and Methods 

### 2.1. Study Design and Population

This study used the secondary data of 2019 Korea Youth Risk Behavior Web-based Survey (KYRBS), a cross-sectional study conducted by the Korea Centers for Disease Control and Prevention (KCDC). A stratified multistage probability sampling was used to produce nationally representative statistics concerning health behaviors of Korean adolescents. Students from grades 7–12 anonymously completed a self-reported online survey. The survey was conducted from June to July 2019, two years after the introduction of HTP in Korea. A total of 57,303 students from 800 sample schools (400 junior high schools and 400 high schools) participated (response rate = 95.3%) [17]. The KYRBS was approved by the Institutional Review Board of the KCDC (2014-06EXP-02-P-A). 

### 2.2. Measures

#### 2.2.1. Tobacco Products Use

The present study examined the use of three types of tobacco products, including CCs, ECs, and HTPs. The participants were asked whether they had ever used the products, and ever users of each tobacco products were asked if they had used them within the past 30 days. Based on their responses, participants were categorized into current, former, or never users, respectively [11,18]. The 2019 KYRBS differed from previous years since it included a new question concerning current HTP use. The questions regarding HTPs were as follows: “Have you ever used HTPs (IQOS^®^, glo^®^ or lil^®^)?” and “Within the past 30 days, how many days did you use HTPs (IQOS^®^, glo^®^ or lil^®^), even just one puff?”. Respondents who had used HTPs within the past 30 days were defined as current HTP users, while those who had never used HTP were classified as never HTP users. Former HTP users were those who had used HTPs but not within the past 30 days. CC or EC users were similarly classified. For ECs, the participants were asked to answer the following questions: “Have you ever used nicotine-containing ECs?” and “Within the past 30 days, how many days did you use nicotine-containing ECs?” Two questions regarding CC use were “Have you ever tried cigarette smoking, even one or two puffs?” and “Within the past 30 days, how many days have you smoked cigarettes?” In addition, current CC or EC users were further classified according to their smoking frequencies by applying the criteria used by Youth Risk Behavior Surveillance System in the United States to classify youth smokers according to smoking frequency: <20 days/month, 20–29 days/month (current frequent cigarette use), and 30 days/month (current daily cigarette use) [19].

#### 2.2.2. Other Characteristics

Covariates included sociodemographic and lifestyle factors, including sex, grade (7–12), residential area (metropolitan, small- or medium-sized city, or rural), stress level (low, moderate, or high), subjective academic performance (high, moderate, or low), perceived economic status (high, moderate, or low), and monthly alcohol consumption (yes or no).

### 2.3. Statistical Analysis

Weighted percentages of former and current HTP users were calculated. Moreover, the prevalence and proportion of poly-tobacco use within the past 30 days were calculated based on weighted percentages. Two different multivariate multinomial logistic regression models were designed to evaluate HTP use according to the following effects: (1) current CC and/or EC use and (2) experience of CC or EC use and smoking frequency. In model 1, HTP use was compared to current use of CC and/or EC. In model 2, HTP use status was compared separately according to experience of CC or EC use and frequency of smoking. Two models were designed because of an increment developed by the differences between prevalence of HTP use—0.1% in non-users and 62.9% in dual users—caused by nine categories considering the use status (current, former, or never) of CCs and ECs. For each model, former and current HTP users were compared with HTP never users (reference group) and former HTP users were compared with current HTP users (reference group). Each model was adjusted for all covariates. All analyses were performed using SAS version 9.4 (SAS Institute Inc., Cary, NC, USA), and a *p*-value < 0.05 was considered statistically significant.

## 3. Results

Characteristics of study participants and their HTP use are shown in Table 1. Of all participants, 2.6% were current HTP users and 2.3% were former HTP users. First, the prevalence of current HTP use according to current CC or EC use was evaluated. The highest prevalence was among current dual users of CC and EC (62.9%), and the lowest was among non-users of CC and EC (0.1%). Second, the prevalence of current HTP users was examined according to the experience of CC or EC use and frequency of smoking. The prevalence of HTP use increased proportionately with increase CC and EC use. The prevalence of current HTP use among current EC users was higher than that of current CC users, and was highest among current daily EC users (73.0%). The highest prevalence of former HTP use among CC and EC users was observed in current daily CC users (22.3%) and former EC users (35.7%). The higher prevalence of former and/or current HTP use was associated with men, upper grades, high stress level, low academic performance and economic status, and monthly alcohol drinkers.

A total of 7.3% of all participants were current users of any tobacco products. Most of them were current CC-only users (46.3%), followed by triple users (22.7%), dual users of CC and EC (13.4%), and dual users of HTP and CC (9.1%). Of all current HTP users, only 4.1% were single users while the proportions of dual and triple users were 63.4% and 32.4%, respectively. A similar pattern was detected among current EC users, but not among CC users (Table 2).

Table 3 illustrates the results of multinomial logistic regression analyses that examined factors related to HTP use. Model revealed that CC-only users (adjusted odds Ratio [AOR] 106.60, 95% confidence interval [CI] 79.62–142.71), EC only users (AOR 355.93, 95% CI 242.16–523.17), and dual users of CC and EC (AOR 885.21, 95% CI 661.49–1184.01) were more likely to be current HTP users than current non-CC and EC users. Similarly, current CC or EC use was associated with former HTP use, but there were no significant differences in AOR values between each group; AORs were 13.75 (95% CI 11.63–16.25), 13.76 (95% CI 9.11–20.79), and 14.62 (95% CI 11.55–18.50) for CC-only, EC-only, and dual users, respectively. AORs for former HTP users currently using CCs or ECs were lower compared to current HTP users, with their lowest AOR (0.02 (95% CI 0.01–0.02)) being among current dual users of CCs and ECs.

Model 2 revealed that AORs for current HTP users increased proportionately with CC or EC smoking frequency. The increment of AORs (by smoking frequency) was greater in current EC daily users (AOR 59.97, 95% CI 38.28–93.92) than in current CC daily users (AOR 31.79, 95% CI 22.67–44.58). Moreover, the likelihood of former HTP use was proportionately associated with CC smoking frequency; this pattern was not observed in EC users, showing the highest AOR, 16.91 (95% CI 13.58–21.05) in former EC users, but not in current EC users. Compared to current HTP users, AORs for former HTP users according to CC or EC use and smoking frequency were high: 8.22 (95% CI 5.23–12.91) in former CC users and 2.34 (95% CI 1.67–3.29) in former EC users. Regarding CC use, there were no statistically significant differences between never users and current users, regardless of smoking frequency. However, former HTP use was inversely proportionate to EC smoking frequency (lowest in those with current daily EC users: AOR 0.18, 95% CI 0.12–0.28 (Table 3).

## 4. Discussion

Our study showed that there was a rapid increase in interest toward HTPs and a high level of dual or triple use pattern of CC or EC. First, the prevalence of ever (former or current) and current HTP users among Korean adolescents was 4.9% and 2.6%, respectively, within two years of the launch of HTPs. Compared to the previous year (2.9%) [13], the prevalence of ever HTP users increased by approximately 1.8 times (2.0% p). Moreover, the 2019 prevalence of current HTP use among adolescents was slightly higher than among adults (2.1%) in 2018 from a study on Korean adults [10]. In addition, although a one year gap existed, the current HTP users among Korean adolescents (2.6%) is approximately half of current HTP use among individuals in their 20s (4.3%) from the aforementioned study [10], the group with the most HTP users [10]. The ratio between CC smoking rates of adolescents (6.7%) and people in their 20s (23.8%) was less than 0.3 in 2018 [20]. However, the ratio between the HTP smoking rates of adolescents and adults aged 20–29 was about 0.5, indicating adolescents’ early exposure to HTPs. Similarly, in Japan—one of the first countries introduced to IQOS^®^—the prevalence of current IQOS^®^ users (in the first year after the launch) aged 15 to 19 years was 0.6%. However, use among the same participants increased to 2.3% one year later [12]. 

Adolescent responses to HTPs are presumably due to two factors. First, tobacco companies’ marketing strategies via social media are designed to appeal to young consumers [8,9,21]. Additionally, companies emphasize the reduction of tobacco smell as an advantage of HTPs [22]. Second, HTPs were introduced in the market shortly after the Korean government increased CC price by 80%, banned smoking in all restaurants, and made pictorial warning labels on cigarette packs mandatory [23]. These factors contributed to the success of IQOS^®^’ marketing strategy. Moreover, this might have influenced HTP use among adolescents. 

The proportion of current HTP users who used CC or EC was surprisingly high (95.9%). This pattern of poly-tobacco use by adolescents was similarly observed in Korean adult HTP user (98.4%) [10]. HTP use was associated to current CC or EC use, especially among dual users. In addition, current HTP users who used CC or EC together were less likely to quit HTPs. Although the association of EC with HTP use among people aged 15 or older has not been evaluated in Japan, the association of current CC use with current HTP use was observed in our study, confirming earlier research [12]. 

The dual use pattern of HTPs and other tobacco products among adolescents differed from that of adults’ regarding frequency of CC smoking. While CC smoking frequency and the likelihood of HTP dual use were proportionately associated among adolescents, a similar trend was not detected among adults [10]. Thus, the likelihood of HTP dual use was directly proportional to CC smoking frequency among adults who smoked less than one pack per day, but it was inversely proportional among adults who smoked more than 20 cigarettes per day [10]. This difference could be because HTPs’ role as a complement to CC might be diminished among adult heavy smokers because their smoking habits are already established, while adolescents’ smoking habits are not, even among daily smokers [24]. Therefore, HTP use among adolescents is a new public health concern, as it can lead to dual or triple use of tobacco products, especially because dual or poly-tobacco use patterns among adolescents have been commonly reported [15,25,26]. However, targeted regulation of certain tobacco products might result in an increase in the use of other tobacco products. For example, strengthening regulation for CC could lead to a trend in HTP use among adolescents. Since HTP use among adolescents is not recommended for any purpose and dual or poly-tobacco use with HTP could pose significant health risks, in order to prevent it, comprehensive tobacco control policies are needed for all tobacco products. 

Regarding the association of HTP use with CCs and ECs, current use of CC was not associated with the possibility of HTP cessation, compared with never CC users. Considering different perceptions of tobacco products among adolescents [27], their intentions and self-defined reasons for using CCs or ECs along with HTPs might vary. Further, ECs and HTPs have been accepted by adolescents as mutually complementary, so their using pattern correlates as either increasing (dual current use) or decreasing (dual former use). This phenomenon could be because HTPs are recognized as a type of ECs in Korea [21]. For example, a widely accepted term of HTP was “cigarette-type EC” in Korea, instead of “heat-not-burn” or “heated tobacco products”, and this makes it difficult to distinguish between ECs and HTPs. Moreover, results showed that current HTP use had a stronger association with current EC use than current CC use. Further, although HTPs are not completely complementary to CCs, HTP is not an alternative for CC, as shown in previous studies with Korean adult [10] because current CC use had no differential impact between current and former HTP users. This is further supported by our study: (1) most HTP users were concurrently using CCs, and (2) current CC users were more likely to be dual users of HTPs. Contrarily, the odds ratio for current HTP use was significantly higher in former CC users than in CC never users, which might suggest that HTP supplements efforts to quit CC use. However, since this study was a cross-sectional study and there was no information available on the association of the timing of smoking cessation with HTP use, it was not possible to understand the causal relationship between them. In addition, the OR value of HTP use among former CC users was much lower than that of current CC users, and most HTP users were dual CC users, which suggests that HTP use has little influence on CC use cessation among adolescents.

The present study has several limitations. First, the causal relationship between HTP use and EC or CC use could not be determined because KYRBS was a cross-sectional survey. Second, the use of tobacco products may have been underestimated since KYRBS is a self-reported survey [28]. It can be presumed that false negative responses about each tobacco product exist, regardless of product type. However, although adolescents tend to conceal their CC smoking experience, they are more likely to respond truthfully about HTP use due to their public acceptance. In such cases, the dual use pattern of HTPs and CCs shown in this study might have been underestimated. Moreover, false negative responses may not meaningfully affect the overall result since the strength of the association of HTP use with CC use was significant.

Despite these limitations, the major strengths of this study include its first examination of the prevalence of current HTP use and the association of other tobacco products with HTP use, analyzing nationally representative data of a large sample of over 57,000 adolescents. Considering the worldwide spread of HTPs, our study results can contribute to the development of tobacco control policies for new tobacco products.

## 5. Conclusions

A total of 2.6% of Korean adolescents were found to be current HTP users, and most of them (95.9%) were dual or triple users of CC or EC. The likelihood of current HTP use was higher among current CC or EC users, especially dual users or those with higher smoking frequency. Our study result suggests that HTPs, along with other tobacco products, might become a public health concern for adolescents. In order to prevent smoking in adolescents, it is essential to establish comprehensive tobacco control policies for various tobacco products.

## Figures and Tables

**Table 1:** Characteristics of participants and heated tobacco products use according to characteristics.

	Full Sample		HTP Use	
	N	Weighted % ^a^	Former User (Weighted %)	Current User ^b^ (Weighted %)
Total	57,303	100.0	2.3%	2.6%
Tobacco products use				
<Classification 1: according to current CC/EC-use>				
Non-users	53,334	92.8	1.2	0.1
CC only	2251	4.0	21.8	16.4
EC only	276	0.5	14.6	36.1
Dual users	1442	2.6	11.0	62.9
<Classification 2: according to CC/EC-use and smoking frequency>				
CC-use				
Never	50,227	87.3	0.2	0.2
Former	3383	6.0	15.7	1.6
Current (<20days/month)	1652	2.9	12.3	21.9
Current (20–29days/month)	331	0.6	18.2	34.7
Current (30days/month)	1710	3.2	22.3	46.7
EC-use				
Never	53,268	92.6	0.5	0.3
Former	2317	4.3	35.7	10.7
Current (<20days/month)	1276	2.3	11.6	54.6
Current (20–29days/month)	112	0.2	13.1	59.6
Current (30days/month)	330	0.6	11.3	73.0
Socio-demographics				
Sex				
Men	29,841	52.0	3.6	4.0
Women	27,462	48.0	1.0	1.2
Grade				
7th	9738	15.9	0.4	0.2
8th	9665	15.3	1.0	1.2
9th	9981	16.6	2.2	1.6
10th	9273	17.1	3.0	3.0
11th	9044	16.5	3.2	4.4
12th	9602	18.5	3.8	4.9
Residential area				
Metropolitan	29,356	50.7	2.3	2.6
small- or medium-sized city	24,380	44.8	2.4	2.7
Rural	3567	4.6	2.1	2.0
Stress level				
High	22,778	39.9	2.5	3.2
Moderate	23,403	41.0	2.3	2.1
Low	11,122	19.1	2.1	2.6
Subjective academic performance				
High	21,943	38.1	1.6	1.9
Moderate	17,234	30.1	2.0	1.9
Low	18,126	31.8	3.5	4.1
Perceived economic status				
High	22,505	39.7	2.2	2.6
Moderate	27,457	47.8	2.2	2.2
Low	7341	12.5	3.2	4.2
Monthly Alcohol consumption				
Yes	48,903	85.0	8.3	13.6
No	8400	15.0	1.3	0.7

^a^ Rows may not be added up to 100% due to rounding. ^b^ Current use of heated tobacco products indicates its use within the past 30 days. Abbreviations: HTP, heated tobacco product; EC, electronic cigarette; CC, conventional cigarette.

**Table 2:** Poly-tobacco use (current use of two or more tobacco products) patterns of three tobacco products among tobacco product users.

	Prevalence (Weighted %)	Proportion (Weighted %) ^a^			
		Total	HTP	EC	CC
Any tobacco products	7.3	100.0	100.0	100.0	100.0
Single users					
HTP only	0.1	1.5	4.1	-	-
EC only	0.3	4.5	-	10.4	-
CC only	3.4	46.3	-	-	50.6
Dual users					
HTP & CC	0.7	9.1	25.3	-	9.9
HTP & EC	0.2	2.5	7.1	5.9	-
CC & EC	1.0	13.4	-	31.1	14.7
Triple users (HTP, EC & CC)	1.7	22.7	63.4	52.6	24.8

^a^ Rows may not be added up to 100% due to rounding. Abbreviations: HTP, heated tobacco product; EC, electronic cigarette; CC, conventional cigarette.

**Table 3:** Evaluation of status of heated tobacco products use according to conventional cigarettes and e-cigarettes use pattern from multinomial logistic regression model.

	Model 1			Model 2		
	Current ^a^ versus Never HTP	Former versus Never HTP	Former versus Current ^a^ HTP	Current ^a^ versus Never HTP	Former versus Never HTP	Former versus Current ^a^ HTP
	AOR (95% CI)	AOR (95% CI)	AOR (95% CI)	AOR (95% CI)	AOR (95% CI)	AOR (95% CI)
Tobacco products use						
<Classification1: according to current CC/EC-use>						
Non-users	Ref	Ref	Ref	-	-	-
CC only	**106.60 (79.62–142.71)**	**13.75 (11.63–16.25)**	**0.13 (0.09–0.18)**	-	-	-
EC only	**355.93 (242.16–523.17)**	**13.76 (9.11–20.79)**	**0.04 (0.02–0.06)**	-	-	-
Dual users	**885.21 (661.49–1184.01** **^b^** **)**	**14.62 (11.55–18.50)**	**0.02 (0.01–0.02)**	-	-	-
<Classification 2: according to CC/EC-use and smoking frequency>						
CC-use						
Never	-	-	-	Ref	Ref	Ref
Former	-	-	-	**1.94 (1.34–2.81)**	**15.93 (12.03–21.09)**	**8.22 (5.23–12.91)**
Current (<20days/month)	-	-	-	**14.16 (10.01–20.01)**	**13.95 (10.15–19.16)**	0.99 (0.62–1.57)
Current (20–29days/month)	-	-	-	**20.47 (12.59–33.28)**	**22.32 (14.22–35.02)**	1.09 (0.62–1.92)
Current (30days/month)	-	-	-	**31.79 (22.67–44.58)**	**34.01 (25.12–46.04)**	1.07 (0.69–1.67)
EC-use						
Never	-	-	-	Ref	Ref	Ref
Former	-	-	-	**7.22 (5.41–9.65)**	**16.91 (13.58–21.05)**	**2.34 (1.67–3.29)**
Current (<20days/month)	-	-	-	**34.54 (25.28–47.18)**	**8.04 (6.01–10.77)**	**0.23 (0.16–0.34)**
Current (20–29days/month)	-	-	-	**37.15 (20.01–68.99)**	**8.81 (4.32–17.96)**	**0.24 (0.12–0.47)**
Current (30days/month)	-	-	-	**59.97 (38.28–93.92)**	**10.68 (6.61–17.25)**	**0.18 (0.12–0.28)**
Sociodemographics						
Sex						
Men	**2.11 (1.77–2.53)**	**3.1 (2.65–3.64)**	**1.47 (1.17–1.85)**	**1.73 (1.41–2.12)**	**1.47 (1.23–1.76)**	0.85 (0.67–1.08)
Women	Ref	Ref	Ref	Ref	Ref	Ref
Grade						
7th	Ref	Ref	Ref	Ref	Ref	Ref
8th	**2.26 (1.21–4.24)**	**1.99 (1.33–2.96)**	0.88 (0.43–1.78)	**2.35 (1.25–4.40)**	1.25 (0.80–1.96)	0.53 (0.26–1.09)
9th	**2.34 (1.27–4.31)**	**3.80 (2.60–5.54)**	1.62 (0.81–3.24)	**2.09 (1.13–3.88)**	**1.57 (1.04–2.36)**	0.75 (0.37–1.51)
10th	**3.23 (1.77–5.89)**	**4.19 (2.89–6.07)**	1.30 (0.66–2.55)	**2.84 (1.54–5.21)**	**1.73 (1.18–2.53)**	0.61 (0.31–1.19)
11th	**4.77 (2.61–8.73)**	**4.06 (2.82–5.84)**	0.85 (0.43–1.66)	**4.18 (2.27–7.70)**	**1.59 (1.09–2.31)**	0.38 (0.20–0.74)
12th	**4.91 (2.71–8.93)**	**4.61 (3.20–6.66)**	0.94 (0.48–1.83)	**3.97 (2.17–7.28)**	**1.72 (1.18–2.51)**	**0.43 (0.22–0.84)**
Area						
Metropolitan)	1.07 (0.72–1.59)	1.10 (0.80–1.51)	1.03 (0.66–1.61)	0.89 (0.58–1.36)	0.87 (0.61–1.25)	0.98 (0.64–1.51)
Small- or medium-sized city	1.07 (0.72–1.59)	1.07 (0.78–1.48)	1.00 (0.64–1.57)	0.93 (0.61–1.42)	0.88 (0.61–1.27)	0.95 (0.61–1.47)
Rural	Ref	Ref	Ref	Ref	Ref	Ref
Stress level						
High	1.00 (0.81–1.22)	1.14 (0.95–1.37)	1.15 (0.90–1.46)	0.95 (0.76–1.19)	0.97 (0.79–1.18)	1.01 (0.79–1.31)
Moderate	0.83 (0.65–1.06)	1.11 (0.95–1.31)	**1.35 (1.02–1.77)**	0.76 (0.58–1.01)	0.98 (0.81–1.19)	1.29 (0.96–1.74)
Low	Ref	Ref	Ref	Ref	Ref	Ref
Subjective academic performance						
High	Ref	Ref	Ref	Ref	Ref	Ref
Moderate	**0.80 (0.64–0.99)**	1.09 (0.91–1.30)	1.37 (1.08–1.74)	0.78 (0.61–1.00)	0.95 (0.77–1.18)	1.22 (0.95–1.57)
Low	0.97 (0.80–1.19)	**1.36 (1.13–1.63)**	1.39 (1.11–1.75)	0.83 (0.65–1.06)	0.93 (0.76–1.14)	1.13 (0.88–1.43)
Perceived economic status						
High	Ref	Ref	Ref	Ref	Ref	Ref
Moderate	**0.82 (0.69–0.97)**	0.90 (0.78–1.03)	1.10 (0.90–1.34)	0.83 (0.69–1.01)	0.93 (0.78–1.10)	1.11 (0.9–1.38)
Low	0.93 (0.75–1.15)	1.00 (0.82–1.21)	1.07 (0.84–1.36)	0.93 (0.73–1.20)	0.97 (0.79–1.20)	1.04 (0.8–1.35)
Monthly Alcohol consumption						
Yes	**2.61 (2.19–3.11)**	**2.54 (2.20–2.94)**	0.98 (0.78–1.21)	**2.42 (1.99–2.94)**	**1.47 (1.27–1.69)**	0.61 (0.49–0.76)
No	Ref	Ref	Ref	Ref	Ref	Ref

Abbreviations: AOR, adjusted odds ratio; CI, confidence interval; HTP, heated tobacco product; EC, electronic cigarette; CC, conventional cigarette. Models 1 and 2 were adjusted for all covariates (sex, grade, residential area, stress level, subjective academic performance, perceived economic status, and monthly alcohol consumption). Bold characters indicate significant associations (*p* < 0.05). ^a^ Current use of Heated tobacco products or e-cigarette indicate its use within the past 30 days. ^b^ Upper limit of confidence interval was not provided (reported as >999.999) so that the figure was obtained by calculation using Wald test: exponential value of (estimate +1.96 standard error).
